# Carbapenem-resistant *Klebsiella pneumoniae* from clinical infections: a multifactorial analysis of resistance, virulence, and biofilm potential

**DOI:** 10.3389/fcimb.2025.1712034

**Published:** 2025-11-24

**Authors:** Ramya Juliet, Ramesh Nachimuthu

**Affiliations:** Antibiotic Resistance and Phage Therapy Laboratory, Centre for Advanced Research in Bacteriophage and Infectious Diseases, School of Bio Sciences and Technology, Vellore Institute of Technology (VIT), Vellore, Tamil Nadu, India

**Keywords:** antimicrobial resistance, biofilms, hypervirulent, hypermucoviscous, *Klebsiella pneumoniae*

## Abstract

**Introduction:**

*Klebsiella pneumoniae* has emerged as a major nosocomial pathogen, with hypermucoviscous and hypervirulent variants contributing to severe clinical outcomes. Understanding the interplay between antimicrobial resistance, virulence determinants, and biofilm formation is essential for effective management.

**Methods:**

A total of 145 clinical isolates of *K. pneumoniae* were evaluated for antimicrobial susceptibility, virulence genes, and biofilm-forming capacity. Disk diffusion and minimum inhibitory concentration (MIC) assays were performed to determine resistance patterns. Polymerase chain reaction (PCR) was used to detect carbapenemase and virulence genes, while hypermucoviscosity was assessed through the string test. Biofilm formation was quantified phenotypically.

**Results:**

Disk diffusion revealed that 73% of isolates were multidrug-resistant. MIC testing showed high resistance to meropenem (71%), colistin (61%), and tigecycline (43%). PCR analysis detected *bla*_NDM_ and *bla*_OXA-48_ in 14% of isolates, including two that co-harbored both genes. Virulence determinants such as *iucA* (aerobactin) and *rmpA* were present in 7% of isolates. Hypermucoviscosity was observed in 10% of isolates by the string test; however, only two of these exhibited strong biofilm formation. Overall, 86% of isolates demonstrated biofilm-forming ability.

**Discussion:**

These findings highlight the convergence of antimicrobial resistance, virulence factors, and biofilm-forming capacity in *K. pneumoniae*. The coexistence of these traits promotes persistence, increases pathogenic potential, and complicates therapeutic interventions, emphasizing the urgent need for strengthened infection control and alternative treatment strategies.

## Introduction

*Klebsiella pneumoniae* (Kp) is a Gram-negative bacterium that normally colonizes mucosal surfaces in humans and animals as part of the commensal microbiota but can act as an opportunistic pathogen, causing pneumonia, urinary tract infections, septicemia, and meningitis (Muraya et al., 2022). It is one of the major ESKAPE pathogens (*Enterococcus faecium, Staphylococcus aureus, Klebsiella pneumoniae, Acinetobacter baumannii, Pseudomonas aeruginosa*, and *Enterobacter* spp.) responsible for hospital-acquired infections worldwide (Wyres and Holt, 2016). Clinical isolates of *K. pneumoniae* frequently exhibit multidrug resistance (MDR) through mechanisms such as β-lactamase production, horizontal gene transfer, efflux pump activity, target-site mutations, porin loss, and biofilm formation (Martin and Bachman, 2018; Li et al., 2023).

*K. pneumoniae’s* remarkable genomic plasticity facilitates the acquisition of novel resistance and virulence determinants, leading to the emergence of distinct pathotypes with enhanced pathogenic potential (Russo and Marr, 2019). Of particular concern is the increasing resistance to last-resort drugs, including carbapenems and colistin (Azam et al., 2021), along with the global spread of hypervirulent (hvKp) and hypermucoviscous (hmKp) strains (Dai and Hu, 2022). First identified in the Asian Pacific Rim in 1986, hvKp strains are now reported worldwide, causing severe community-acquired infections and invasive diseases in otherwise healthy individuals (Russo et al., 2014). These strains often exhibit strong biofilm-forming ability, enhanced virulence, and the capacity to disseminate from the primary infection site (Kocsis, 2023).

Classical *K. pneumoniae* (cKp) has been associated with healthcare-associated infections, particularly in immunocompromised patients. However, novel variants, including multidrug-resistant (MDR-Kp), carbapenem-resistant (CR-Kp), hypervirulent (hvKp), and hypermucoviscous (hmKp) strains, have emerged as major causes of both hospital- and community-acquired infections. The global rise of hvKp over the past three decades is concerning, with mortality rates ranging from 3–31% and up to 35% in hvKp-associated bacteremia (Russo and Marr, 2019; Li et al., 2018).

Virulence in *K. pneumoniae* is mediated by multiple factors, including fimbriae, polysaccharide capsules, lipopolysaccharides, and siderophores, with hypervirulent strains further distinguished by traits such as hypercapsulation, increased siderophore production, and exopolysaccharide secretion (Wang et al., 2020). Importantly, hypervirulence and hypermucoviscosity represent related yet distinct characteristics. Hypermucoviscosity is a phenotypic manifestation associated with increased capsule production, whereas hypervirulence is attributed to the coordinated expression of siderophores and other virulence regulators. Notably, not all hvKp isolates exhibit hypermucoviscosity, and vice versa (Choby et al., 2019). Recognizing the public health threat posed by these strains, the World Health Organization has emphasized the necessity for integrated surveillance targeting both resistance and virulence determinants (WHO, 2024).

In this study, we aimed to comprehensively characterize clinical *K. pneumoniae* isolates from Chennai, India, with particular emphasis on antimicrobial resistance to last-resort agents. Antimicrobial susceptibility profiles were determined to categorize isolates as MDR, non-MDR, or susceptible, and strains were further examined for key carbapenemase genes to define the underlying resistance mechanisms. In parallel, major virulence determinants, including exopolysaccharide production and biofilm-forming ability, were evaluated using complementary phenotypic and genotypic approaches. By comparing these traits across resistance categories, the study seeks to elucidate associations between carbapenem resistance, virulence factors, and biofilm formation, thereby contributing to a better understanding of the pathogenic potential and clinical risk posed by *K. pneumoniae*.

## Materials and methods

### Ethical approval

Ethical approval was obtained from the Institutional Ethics Committee for Studies on Human Subjects (IECH), reference number: no. VIT/IECH/2024/15 IECH/15 June 2024/5.

### Collection of bacterial isolates

**Figure 1:**
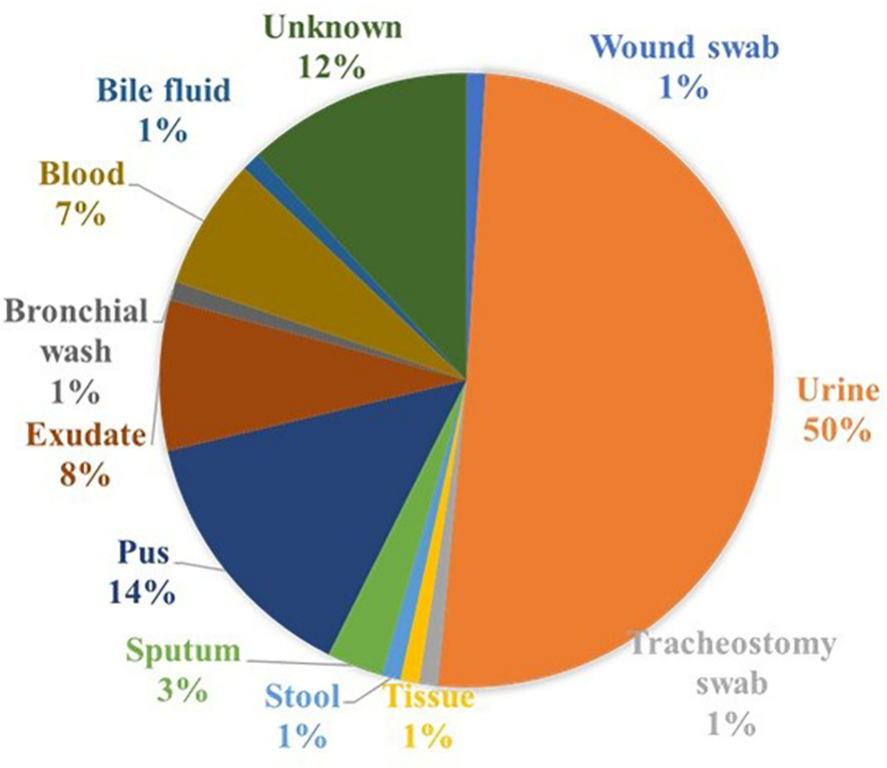
Schematic representation of the distribution of *K. pneumoniae* isolate sources.

A total of 145 non-duplicate *K. pneumoniae* isolates were obtained between 2021 and 2024 from diverse clinical specimens, including urine (n = 74), blood (n = 11), pus (n = 21), exudates (n = 11), and other sample types (n = 28) at a diagnostic center in Chennai, India (**Figure 1**). Preliminary identification was performed based on colony morphology on MacConkey agar (HiMedia, India), where isolates typically appeared as large, mucoid, pink to red colonies. Species-level confirmation was achieved using the VITEK 2 automated system (bioMérieux). All isolates were preserved in cryoprotectant vials before subsequent phenotypic and genotypic analyses.

### Antimicrobial susceptibility test

The antimicrobial resistance profiles of the clinical *K. pneumoniae* isolates were evaluated using the Kirby–Bauer disk diffusion method and interpreted in accordance with CLSI guidelines (CLSI, 2025). The antibiotics tested included meropenem (MRP) (10 µg), imipenem (IPM) (10 µg), ertapenem (ETP) (10 µg), ceftazidime (CAZ) (30 µg), cefotaxime/clavulanic acid (CEC) (30/10 µg), cefixime (CFM) (5 µg), ceftriaxone (CTR) (30 µg), gentamicin (GEN) (10 µg), amikacin (AK) (10 µg), piperacillin/tazobactam (PIT) (100/10 µg), ciprofloxacin (CIP) (30 µg), and levofloxacin (LE) (5 µg) (HiMedia, India). Isolates exhibiting resistance to at least one agent in three or more antimicrobial classes were categorized as multidrug-resistant (Cosentino et al., 2023).

### Minimum inhibitory concentration determination

MIC determination was carried out for meropenem, colistin, and tigecycline (Sigma Aldrich, India), as these antibiotics are considered last-resort agents for the treatment of MDR *K. pneumoniae*, using the microbroth dilution method following established protocols (Manohar et al., 2021). Bacterial suspensions adjusted to 0.5 McFarland were inoculated into 96-well plates containing two-fold serial dilutions of each antibiotic (0.25–128 µg/mL) in cation-adjusted Mueller-Hinton broth (HiMedia, India) and incubated at 37°C for 16–18 h. *Escherichia coli* ATCC 25922 was included as a quality control strain. MIC values were interpreted according to CLSI guidelines (CLSI, 2025).

To assess antimicrobial activity across the isolate collection, MIC_50_ and MIC_90_ values were calculated, representing the concentrations that inhibit 50% and 90% of isolates, respectively. Calculations were performed as previously described (Schwarz et al., 2010) using the following formulas:

- MIC_50_ = n x 0.5 (if n is an even); (n+1) x 0.5 (if n is an odd)
- MIC_90_ = n x 0.9

where *n* represents the total number of isolates.

### String test

Hypermucoviscosity was detected using the string test (Kochan et al., 2023). An overnight colony grown on a Luria-Bertani plate (HiMedia, India) was gently touched with a sterile inoculation loop and then lifted vertically. The formation of a viscous string measuring ≥5 mm was considered positive for the hypermucoviscous phenotype, whereas colonies producing strings <5 mm or none were classified as non-hypermucoviscous.

### Congo red agar test

*K. pneumoniae* isolates were streaked onto Brain Heart Infusion (BHI) agar (HiMedia, India) with 5% sucrose and 0.8% Congo red, then incubated overnight at 37°C. Isolates forming black colonies were classified as exopolysaccharide producers based on colony morphology, while others were non-exopolysaccharide producers (Vuotto et al., 2017).

### Quantitative biofilm production assay

Biofilm formation was assessed by the crystal violet assay. Cultures (0.5 McFarland) were diluted 1:10 in Luria Bertani broth (HiMedia, India), and 100 µL was added to 96-well plates, incubated at 37°C for 24 h. Wells were washed with 1x PBS (pH 7.4), air-dried, stained with 0.1% crystal violet for 15 min, then washed again. The stain was solubilized with 33% glacial acetic acid, and OD_570_ was measured (Juliet et al., 2024). Tests were done in triplicate. OD cutoff (ODc) was determined using a blank, and isolates were classified by OD values (Loganathan and Nachimuthu, 2022).

- Non-adherent: OD ≤ OD_c_
- Weak biofilm producers: OD_c_ < OD ≤ 2 OD_c_
- Moderate biofilm producers: 2 OD_c_ < OD ≤ 4 OD_c_
- Strong biofilm producers: OD > 4 OD_c_

### Microscopic examination of biofilm

Three isolates (representing each of strong, moderate, and weak biofilm producers) were analyzed by scanning electron microscopy (SEM). A sterile coverslip in a 12-well plate with 4 mL LB broth and 400 µL overnight culture was incubated at 37°C for 24 h. After washing with 1x PBS (pH 7.4), the coverslip was fixed overnight in 2.5% glutaraldehyde and dehydrated with increasing ethanol concentrations (50–100%). Biofilms were visualized using SEM (CARL ZEISS EVO 18, VIT Vellore) (Vuotto et al., 2017).

### DNA isolation

Bacterial DNA was extracted by the boiling lysis method. Overnight cultures were centrifuged (8000×g, 15 min), pellets resuspended in 100 µL sterile water, and boiled at 95°C for 10 min. After centrifugation (8000×g, 5 min), the supernatant was used as the PCR template (Manohar et al., 2021).

### Screening of resistant and virulence genes

Carbapenem resistance in the clinical isolates was determined using multiplex PCR targeting *bla*_KPC_*, bla*_IMP_*, bla*_VIM_*, bla*_NDM_, and *bla*_OXA-48-like_, genes with an annealing temperature of 60 °C (initial denaturation of 10 min at 95°C; 35 cycles of 45 sec, 95°C; 45 sec, 60°C; 60 sec, 72°C; followed by final extension of 8 min, 72°C) (Manohar et al., 2021). PCR screening targeted the *rmpA* and *iucA* (aerobactin) genes, which are representative markers of hypermucoviscosity and siderophore-mediated virulence, respectively with conditions of 35 cycles, 95°C for 1 min, 50°C for 1 min, and 72°C for 2 min, and a final extension of 72°C for 7 min (Lin et al., 2014). The primers (final concentration- 0.2µM) used are listed in **Supplementary Table S1**. Amplified gene products were sequenced, gene identity was confirmed using BLAST analysis and sequences are submitted in NCBI database (https://www.ncbi.nlm.nih.gov/).

### Statistical analysis

Chi-square analysis was performed to evaluate phenotypic and genotypic associations with MDR, non-MDR, and sensitive *K. pneumoniae* isolates using GraphPad Prism version 8.0.2. A *P* value < 0.05 was considered statistically significant.

## Results

### Phenotypic resistance to antimicrobials

**Figure 2:**
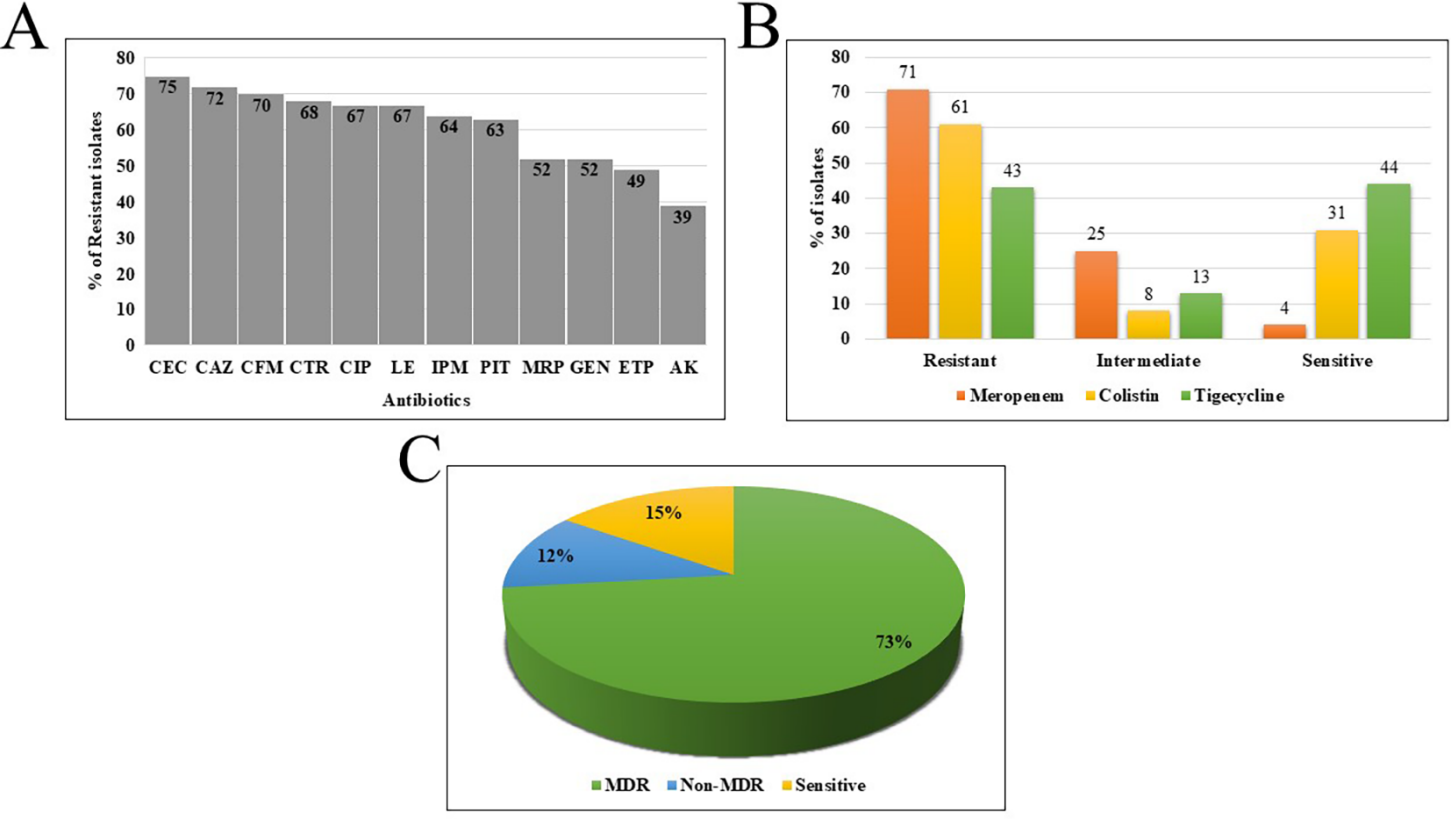
**(A)** Disk diffusion results are represented as the percentage of isolates showing resistance to various antimicrobial agents in descending order. **(B)** Graphical representation of the sensitivity patterns based on minimum inhibitory concentration (MIC) results. **(C)** Pie chart representing the classification of isolates into multidrug-resistant (MDR), non-MDR, and sensitive categories.

Disk diffusion testing showed the highest resistance to cephalosporins: cefotaxime/clavulanic acid (75%), ceftazidime (72%), cefixime (70%), and ceftriaxone (68%). High resistance was also seen in fluoroquinolones (ciprofloxacin and levofloxacin at 67%), imipenem (64%), piperacillin-tazobactam (63%), meropenem (52%), gentamycin (52%), and ertapenem (49%). The lowest resistance was to amikacin (39%) (**Figure 2A**). Susceptibility testing revealed the highest sensitivity for amikacin (53%), followed by ertapenem (45%), gentamycin (44%), and meropenem (36%). Based on these profiles, 106 isolates (73%) were MDR, 17 (12%) were resistant to one or two agents (non-MDR), and 22 (15%) were fully susceptible (**Figure 2C**).

MIC analysis showed 71% (103/145) of isolates were resistant to meropenem, classifying them as carbapenem-resistant *K. pneumoniae* (CR-Kp). Resistance to colistin and tigecycline was found in 61% (88/145) and 43% (62/145) of isolates, respectively (**Figure 2B**). The MIC_50_ and MIC_90_ values were 4 mg/L and 32 mg/L for meropenem, 8 mg/L and 128 mg/L for colistin, and 2 mg/L and 64 mg/L for tigecycline. Notably, 21% (30/145) of isolates were resistant to all three drugs. Dual resistance patterns included meropenem-colistin (21%, 31/145), colistin-tigecycline (10%, 14/145), and meropenem-tigecycline (8%, 12/145). The detailed MIC distribution for meropenem, colistin and tigecycline is provided in **Supplementary Figure S1**. The longitudinal sampling (2021-2024) allowed assessment of temporal trends in antimicrobial resistance. The prevalence of carbapenem-resistant strains fluctuated across the study period, with the highest rates recorded in 2021 (84%) and 2023 (82%), followed by a decline in 2022 (60%) and 2024 (60%) (**Supplementary Table S5**).

### Mucoid phenotype

**Figure 3:**
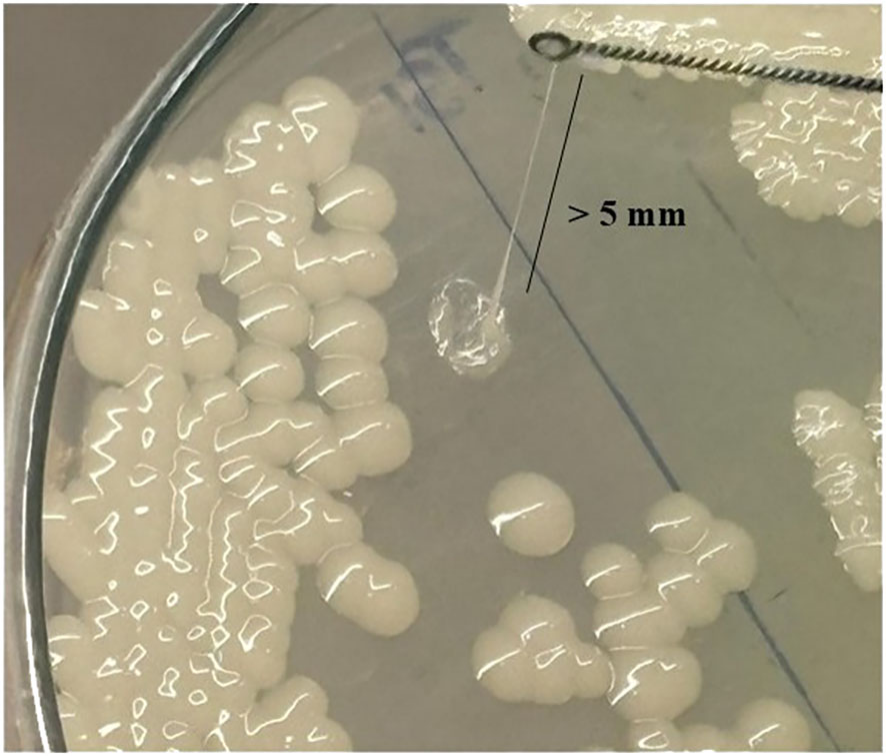
A hypermucoviscous strain showing a string length greater than 5 mm.

Among the 145 isolates, 14 (10%) were positive for the string test, producing viscous strings greater than 5 mm in length (**Figure 3**). Of the string test-positive isolates, 42% (6/14) were MDR, 15% (2/14) were non-MDR, and 43% (6/14) were sensitive. These 14 isolates were thus characterized to be exhibiting a hypermucoviscous phenotype, a prominent characteristic associated with hvKp strains (**Table 1**). A significant association was observed between hypermucoid phenotype and MDR status (χ² = 9.854, df = 2, P = 0.007), indicating that MDR isolates were more likely to be hypermucoid.

**Table 1:** Resistance and virulence patterns of string test positive isolates in association with exopolysaccharide and biofilm production.

Isolate ID	Source	Resistant pattern	Virulence gene	String test	Congo red	Biofilm
*K. pneumoniae* KP4	Sputum	Sensitive	Aerobactin, *rmpA*	+	+	Moderate
*K. pneumoniae* KP9	Pus	MDR	Absent	+	+	Moderate
*K. pneumoniae* KP12	Urine	Non-MDR	Aerobactin, *rmpA*	+	+	Moderate
*K. pneumoniae* KP30	Urine	MDR	Aerobactin, *rmpA*	+	+	Moderate
*K. pneumoniae* KP45	Urine	Non-MDR	Aerobactin, *rmpA*	+	+	Weak
*K. pneumoniae* KP66	Blood	Sensitive	Aerobactin, *rmpA*	+	+	Weak
*K. pneumoniae* KP115	Unknown	Sensitive	Aerobactin, *rmpA*	+	+	Moderate
*K. pneumoniae* KP119	Urine	Sensitive	Absent	+	+	Moderate
*K. pneumoniae* KP121	Pus	MDR	Absent	+	+	Weak
*K. pneumoniae* KP122	Urine	MDR	Absent	+	+	Moderate
*K. pneumoniae* KP137	Unknown	Sensitive	Absent	+	+	Strong
*K. pneumoniae* KP138	Unknown	Sensitive	Absent	+	+	Moderate
*K. pneumoniae* KP144	Urine	MDR	Absent	+	+	Moderate
*K. pneumoniae* KP145	Urine	MDR	Absent	+	+	Strong

‘+’ indicates a positive result for the string test and Congo red agar test.

### Exopolysaccharide production

In the Congo red agar assay, 86 out of 145 isolates exhibited dark red colonies with black pigmentation, indicating elevated exopolysaccharide production, a key component in the biofilm structure. Among these exopolysaccharide-producing isolates, 65% (56/86) were classified as MDR, 16% (14/86) as non-MDR, and 19% (16/86) as sensitive. Notably, all 14-string test-positive isolates were also identified as strong producers of exopolysaccharides. A chi-square test revealed a significant association between exopolysaccharide-producing isolates and antimicrobial resistance profiles (χ² = 7.226, df = 2, P = 0.027), indicating that EPS-positive isolates were more likely to be multidrug-resistant.

### Biofilm quantification

Based on the mean optical density (OD) values, 29 of 145 isolates (20%) were classified as strong biofilm producers. Among these, 22 exhibited black-pigmented colonies in the Congo red assay, demonstrating strong exopolysaccharide production. Additionally, 66 of 145 isolates (46%) were found to be moderate biofilm producers, with 47 testing positive for exopolysaccharide production. Of the 31 isolates (21%) classified as weak biofilm producers, 15 produced black colonies, while among the 19 non-adherent isolates (13%), 2 exhibited exopolysaccharide production on Congo red agar.

**Figure 4:**
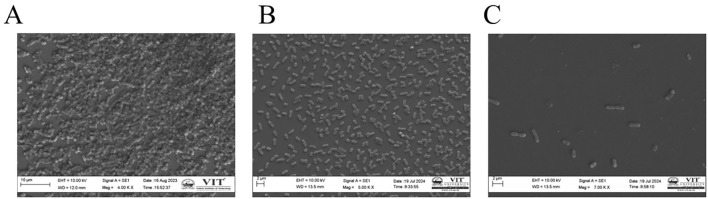
Ultrastructural examination of biofilm formation by three isolates representing **(A)** strong, **(B)** moderate, and **(C)** weak biofilm producers.

Regarding antimicrobial susceptibility profiles, among the 106 MDR isolates, 23% (24/106) were strong biofilm producers, 40% (43/106) were moderate biofilm producers, 22% (23/106) were weak producers, and 15% (16/106) were non-adherent. Among the 17 non-MDR isolates, 2/17 (12%), 11/17 (65%), and 3/17 (17%) were strong, moderate, and weak biofilm producers, and 1/17 (6%) were non-adherent. Out of 22 sensitive isolates, 3/22 (14%) isolates exhibited strong biofilm, 12/22 (54%) exhibited moderate biofilm, 5/22 (23%) exhibited weak biofilm, and 2/22 (9%) were non-adherent. No significant association was observed between biofilm formation strength (strong, moderate, weak, non-adherent) and antimicrobial resistance profiles (χ² = 5.134, df = 6, P = 0.527), indicating that biofilm production was independent of MDR status. Among the 103 carbapenem-resistant *K. pneumoniae* (CR-Kp) isolates identified by MIC testing, 19% (20/103) were strong biofilm producers, 46% (47/103) were moderate, 24% (25/103) were weak producers, and 11% (11/103) were non-adherent. Analysis of the hmKp isolates, 2 of 14 were strong biofilm producers, 9 were moderate producers, and 3 were weak producers, whereas none of the hmKp isolates were non-adherent. SEM analysis revealed clear structural differences among the isolates. The strong biofilm producer KP145 strain (**Figure 4A**) exhibited dense cell aggregation embedded in a thick extracellular matrix, forming a compact multilayered structure. The moderate producer KP144 strain (**Figure 4B**) showed moderate cell clustering with partial surface coverage, while the weak producer KP121 strain (**Figure 4C**) displayed sparse cell attachment with minimal extracellular material and poor surface adherence.

### Detection of carbapenemase and virulence genes

A total of 145 isolates were subjected to genotypic screening for carbapenemase genes, of which 21 isolates (14%) harbored one or more resistance genes. All 21 isolates were found to be MDR. Among these, 14 isolates carried the *bla*_OXA-48 like_ gene, and 9 isolates carried the *bla*_NDM_ gene. Notably, 2 of the 21 isolates co-harbored both *bla*_OXA-48 like_ and *bla*_NDM_ genes (**Table 2**). Sequence analysis confirmed that all *bla*_OXA-48 like_ corresponded to *bla*_OXA-181,_ and all *bla*_NDM_ genes aligned with *bla*_NDM-1_ sequences based on family-specific primers. No other β-lactamase genes were detected in this study. No significant association was observed between the presence of carbapenemase genes and multidrug resistance, with *bla*_NDM_ (χ² = 3.530, df = 2, P = 0.171) and *bla*_OXA-48-like_ (χ² = 5.701, df = 2, P = 0.058). None of the resistance gene-carrying isolates exhibited a positive string test. In antimicrobial susceptibility testing, all carbapenemase gene-positive isolates were resistant to meropenem by disk diffusion. By MIC testing, all but one isolate carrying the *bla*_OXA-48 like_ gene were resistant to meropenem. Among the 14 *bla*_OXA-48 like_ positive isolates, 2 were strong, 9 were moderate, and 3 were weak biofilm producers. Among the 9 *bla*_NDM_ positive isolates, 4 exhibited moderate biofilm production, 3 were weak, and 2 were non-adherent.

**Table 2:** Minimum inhibitory concentrations of meropenem in β-lactamase gene–positive *K. pneumoniae* isolates.

Isolate ID	Gene	Meropenem MIC (μg/mL) ≥ 4 -Resistant
KP13	*bla*_OXA-48 like_	128
KP26	*bla*_OXA-48 like_	4
KP35	*bla*_OXA-48 like,_ *bla*_NDM_	32
KP36	*bla*_OXA-48 like_	32
KP37	*bla*_OXA-48 like_	32
KP38	*bla*_NDM_	64
KP39	*bla*_OXA-48 like_	2
KP41	*bla*_OXA-48 like_	128
KP42	*bla*_NDM_	8
KP43	*bla*_OXA-48 like,_ *bla*_NDM_	32
KP49	*bla*_OXA-48 like_	8
KP53	*bla*_NDM_	16
KP54	*bla*_NDM_	16
KP56	*bla*_NDM_	32
KP72	*bla*_NDM_	8
KP74	*bla*_NDM_	4
KP90	*bla*_OXA-48 like_	4
KP106	*bla*_OXA-48 like_	4
KP110	*bla*_OXA-48 like_	4
KP111	*bla*_OXA-48 like_	4
KP127	*bla*_OXA-48 like_	8

Screening for virulence genes revealed the co-presence of *rmpA* and aerobactin genes in 10 isolates. Among these, 6 isolates tested positive for the string test, highlighting the association of the hypermucoviscous phenotype with hypervirulent genotypic characteristics. Of the 10-virulence gene-positive isolates, 3 were MDR, 2 were non-MDR, and 5 were sensitive. Despite their virulence potential, all hypervirulent isolates exhibited resistance to at least one last-resort antimicrobial agent (either colistin or meropenem) based on MIC testing. None of the virulence gene-positive isolates were strong biofilm producers; 6 were moderate, 3 were weak biofilm producers, and 1 was non-adherent. Accession numbers for all resistance and virulence genes identified in this study are available in **Supplementary Tables S2**-**S4**. A consolidated overview of antimicrobial resistance patterns, virulence phenotypes, and gene profiles is presented in **Table 3** to facilitate a clearer interpretation of the comparative results.

**Table 3:** Overview of key phenotypic and genotypic characteristics of 145 clinical *K. pneumoniae* isolates included in this study.

Parameter	Category	MDR (n)	Non-MDR (n)	Sensitive (n)	Total (n)	Findings
Antimicrobial susceptibility pattern (Disk diffusion)		106	17	22	145	Highest resistance to cephalosporins (68–75%), fluoroquinolones (67%), and imipenem (64%); lowest to amikacin (39%)
MIC-based resistance	Meropenem	74	10	19	103	Meropenem: 71% Colistin: 61% Tigecycline: 43% Triple-drug resistance: 30 (21%)
	Colistin	75	6	7	88	
	Tigecycline	47	4	11	62	
Hypermucoviscosity (string test)	Positive	6	2	6	14	10% positive; observed in both MDR and susceptible isolates
Exopolysaccharide production (CRA)	Positive	56	14	16	86	59% positive All hvKp (string-positive) isolates showed strong EPS
Biofilm	Strong	24	2	3	29	Strong/moderate biofilms more frequent among MDR isolates; virulence-positive strains not necessarily strong biofilm producers
	Moderate	43	11	12	66	
	Weak	23	3	5	31	
	Non-biofilm	16	1	2	19	
Carbapenemase genes	*bla*_NDM_	9	0	0	9	14% positive, including 2 co-harboring both genes
	*bla*_OXA-48like_	14	0	0	14	
Virulence genes	Aerobactin	10	0	0	10	None co-harbored both virulence and resistance genes
	*rmp*A	10	0	0	10	

## Discussion

MDR-Kp has become a critical concern for healthcare providers, as effective antimicrobial agents are often lacking for treatment (Kot et al., 2023). A study by Pitout et al. (2015) reported that 20–80% of *K. pneumoniae* clinical isolates are resistant to first-line agents such as cephalosporins, aminoglycosides, fluoroquinolones, and trimethoprim-sulfamethoxazole (Pitout et al., 2015). Although previous studies from Chennai (Gowrinathan et al., 2025) have reported high MDR rates, biofilm prevalence, and detection of resistant and virulent genes, our study extends these findings by integrating longitudinal sampling from 2021–2024 with comprehensive phenotypic and genotypic characterization. By employing three complementary biofilm assays alongside targeted resistance and virulence gene profiling, we provide a more detailed assessment of trends in *K. pneumoniae* resistance and virulence that were not been much explored across multidrug-resistant and sensitive isolates.

This study tested five classes of antimicrobial agents, including carbapenems, cephalosporins, aminoglycosides, fluoroquinolones, and penicillin. The resistance pattern revealed that 73% of isolates were multidrug-resistant, similar to a multicentric study by Wang et al., which showed a higher prevalence of MDR and XDR (extensively drug-resistant) strains (Wang et al., 2023). According to the critical priority pathogen list by the Indian Council of Medical Research (ICMR), the World Health Organization (WHO, India), and the Department of Biotechnology (DBT), carbapenem-resistant, tigecycline-resistant, and colistin-resistant *E. coli* and *K. pneumoniae* are categorized as critical priority pathogens (Department of Biotechnology, Government of India and World Health Organization Country Office for India, 2019).

Resistance to last-resort drugs such as carbapenem, colistin, and tigecycline was assessed through minimum inhibitory concentration. High susceptibility was observed to tigecycline (44%), followed by colistin (31%). Similar to the findings of Azam et al. (2021), co-resistance to tigecycline, meropenem, and colistin was found in 21% of the isolates (Azam et al., 2021). Elevated MIC_50_ and MIC_90_ values for meropenem align with the 71% phenotypic resistance in MIC. Similarly, elevated levels of colistin are also concerning, as it is used as a last-line therapy. The relatively high MIC_50_ and MIC_90_ values for tigecycline emphasize the challenge in managing MDR-Kp infections. The observed variation in resistance frequency between disk diffusion and MIC testing likely arises from methodological differences. Disk diffusion provides a qualitative, zone-based estimate of susceptibility, which may not capture borderline or heteroresistant isolates. MIC testing, being quantitative, offers higher precision and is the reference standard for detecting true resistance. Variations in diffusion rate, agar depth, or inoculum density can further influence disk diffusion results, explaining the slight discrepancies observed between the two methods (Gajic et al., 2022).

The observed year-to-year changes in carbapenem and multidrug resistance among *K. pneumoniae* clinical isolates can most likely be due to the changing landscape of stewardship programs, infection prevention strategies, and prescribing behaviors. Temporary increases in 2021–2022 could potentially be attributed to pandemic-related antibiotic usage (CDC, 2022). These findings underline the complexity and evolution of antibiotic resistance, as well as the importance of ongoing surveillance and integrated stewardship initiatives in limiting the spread of resistant *K. pneumoniae* strains. One major mechanism of carbapenem resistance is the production of carbapenemase enzymes, which hydrolyze β-lactam antibiotics and reduce susceptibility to treatment (Pitout et al., 2015). In this study, the class B beta-lactamase gene *bla*_NDM_ (New Delhi metallo-β-lactamase) was present in 9 isolates, while the class D *bla*_OXA-48 like_ gene was present in 14 isolates. Two isolates harbored both *bla*_NDM_ and *bla*_OXA-48 like_. Interestingly, while MIC results showed 71% resistance to meropenem, only 14% of isolates carried carbapenemase genes.

Capsule production is a key virulence factor in *K. pneumoniae*, helping the bacterium evade host immune defenses such as phagocytosis. This capsule also facilitates bacterial adhesion to mucosal surfaces, enhancing the potential for infection (Clegg and Murphy, 2017). However, distinguishing hvKp and hmKp strains from classical *K. pneumoniae* strains remains challenging in clinical settings. While phenotypic methods, such as the string test, can aid in differentiation, molecular identification of virulence-associated genes is more reliable. In this study, 14 isolates tested positive for the string test and exhibited exopolysaccharide production, though not all of these isolates carried virulence genes or formed strong biofilms. This highlights that, while hypermucoviscosity is a key feature of hvKp isolates, phenotypic tests like the string test cannot fully differentiate hvKp from classical strains.

Biofilm formation by *K. pneumoniae* creates a protective barrier, hindering the penetration of antimicrobial agents. These biofilms consist of bacterial cells embedded in extracellular polymeric substances and are closely associated with capsule production (Donlan, 2002). However, capsule production can mask type-3 fimbriae, key adhesion molecules involved in biofilm formation, limiting the ability of hypermucoviscous isolates to form strong biofilms (Vuotto et al., 2017). In the biofilm assay conducted in this study, 87% of isolates were able to form biofilms, with the majority being moderate biofilm producers.

Iron is essential for bacterial metabolism, particularly in the host environment. Siderophores, such as yersiniabactin, enterobactin, salmochelin, and aerobactin, play a crucial role in iron acquisition and the establishment of infections (Page, 2019; Clegg and Murphy, 2017). While yersiniabactin and aerobactin are more commonly found in hvKp isolates, the latter is more prevalent (Russo et al., 2014). The regulation of hypermucoviscosity in *K. pneumoniae* is mediated by the *rmpA* gene, which enhances capsule production (Hsu et al., 2011). In this study, 10 isolates co-harbored the aerobactin and *rmpA* genes. However, none co-harbored both virulence and resistance genes.

### Therapeutic implications

The coexistence of *bla*_NDM_ and *bla*_OXA-48, like_ in *K. pneumoniae*, represents a major therapeutic challenge, as these enzymes confer resistance to carbapenems. Current treatment options are largely confined to colistin, tigecycline, and ceftazidime–avibactam, although emerging resistance to the former two agents further limits efficacy. Combination regimens such as ceftazidime–avibactam plus aztreonam may offer improved activity against metallo-β-lactamase producers (Isler et al., 2022; Słabisz et al., 2024), while meropenem–vaborbactam and imipenem–relebactam demonstrate potential against OXA-48-like enzymes (Bonnin et al., 2022; Tiseo et al., 2024). Given these constraints, optimizing antibiotic stewardship, infection control, and rapid molecular diagnostics remains essential to guide targeted therapy and contain dissemination of these high-risk clones.

### Limitations of the study

While this study provides a comprehensive analysis of 145 clinical *K. pneumoniae* isolates, certain aspects could be expanded in future research. The isolates were collected from a single geographic location, so findings may be most directly applicable to this region. Additionally, molecular typing or serotyping was not performed, limiting insights into clonal relationships and broader epidemiological comparisons. Finally, the study focused on selected resistance and virulence genes, and further screening could provide a more complete understanding of pathogenic potential. Despite these considerations, the current work offers valuable insights into antimicrobial resistance, virulence determinants, and biofilm formation in multidrug-resistant *K. pneumoniae* isolates.

## Conclusion

The emergence of variants in *Klebsiella pneumoniae* has posed serious health risks in healthcare-associated and community-acquired infections. This study depicts the prevalence of multidrug-resistant strains in clinical settings. The MIC values of the carbapenem genes *bla*_NDM_ and *bla*_OXA-48 like_ carrying isolates were higher. Hypervirulence analysis showed that not all string test-positive isolates contained aerobactin and *rmpA* genes, whereas all the virulence gene-containing isolates tested positive for the string test. These findings highlight the importance of genotypic screening over phenotypic tests. The study emphasizes the heterogeneous nature of *K. pneumoniae* resistance, biofilm formation, and virulence, which allows us to gain a clearer insight into the nature of *K. pneumoniae* infections and to plan a better treatment approach.

## Data Availability

The datasets presented in this study can be found in online repositories. The names of the repository/repositories and accession number(s) can be found in the article/**Supplementary Material**.
