# Correction: Carbapenem-resistant *Klebsiella pneumoniae* from clinical infections: a multifactorial analysis of resistance, virulence, and biofilm potential

**DOI:** 10.3389/fcimb.2025.1762026

**Published:** 2026-01-12

**Authors:** Ramya Juliet, Ramesh Nachimuthu

**Affiliations:** Centre for Advanced Research in Bacteriophage and Infectious Diseases, School of Bio Sciences and Technology, Vellore Institute of Technology (VIT), Vellore, Tamil Nadu, India

**Keywords:** antimicrobial resistance, biofilms, hypervirulent, hypermucoviscous, *Klebsiella pneumoniae*

A correction has been made to the section **Introduction**, Paragraph 3. The following should be removed as the information repeats content already described in **Introduction**, Paragraph 2 ‘These strains have been implicated in invasive diseases such as liver abscesses and bacteremia, even in otherwise healthy individuals (Zhu et al., 2021)’

“Classical *K. pneumoniae* (cKp) has been associated with healthcare-associated infections, particularly in immunocompromised patients. However, novel variants, including multidrug-resistant (MDR-Kp), carbapenem-resistant (CR-Kp), hypervirulent (hvKp), and hypermucoviscous (hmKp) strains, have emerged as major causes of both hospital- and community-acquired infections. The global rise of hvKp over the past three decades is concerning, with mortality rates ranging from 3–31% and up to 35% in hvKp-associated bacteremia (Russo and Marr, 2019; Li et al., 2018).”

A correction has been made to the section **Introduction**, Paragraph 4. The phrase “Carbapenem resistant” was removed because all isolates were screened for key carbapenemase genes, not only the carbapenem-resistant ones. Keeping the phrase would inaccurately imply selective screening.

“Virulence in *K. pneumoniae* is mediated by multiple factors, including fimbriae, polysaccharide capsules, lipopolysaccharides, and siderophores, with hypervirulent strains further distinguished by traits such as hypercapsulation, increased siderophore production, and exopolysaccharide secretion (Wang et al., 2020). Importantly, hypervirulence and hypermucoviscosity represent related yet distinct characteristics. Hypermucoviscosity is a phenotypic manifestation associated with increased capsule production, whereas hypervirulence is attributed to the coordinated expression of siderophores and other virulence regulators. Notably, not all hvKp isolates exhibit hypermucoviscosity, and vice versa (Choby et al., 2019). Recognizing the public health threat posed by these strains, the World Health Organization has emphasized the necessity for integrated surveillance targeting both resistance and virulence determinants (WHO, 2024).

In this study, we aimed to comprehensively characterize clinical *K. pneumoniae* isolates from Chennai, India, with particular emphasis on antimicrobial resistance to last-resort agents. Antimicrobial susceptibility profiles were determined to categorize isolates as MDR, non-MDR, or susceptible, and strains were further examined for key carbapenemase genes to define the underlying resistance mechanisms. In parallel, major virulence determinants, including exopolysaccharide production and biofilm-forming ability, were evaluated using complementary phenotypic and genotypic approaches. By comparing these traits across resistance categories, the study seeks to elucidate associations between carbapenem resistance, virulence factors, and biofilm formation, thereby contributing to a better understanding of the pathogenic potential and clinical risk posed by *K. pneumoniae*.”

A correction has been made to the section **Materials and Methods**, *Collection of Bacterial Isolates*. This sentence “A consolidated overview of antimicrobial resistance patterns, virulence phenotypes, and gene profiles is presented in Table 1 to facilitate a clearer interpretation of the comparative results” was shifted to the **Results** section during proofreading, but was mistakenly not removed from **Methods**.

“A total of 145 non-duplicate *K. pneumoniae* isolates were obtained between 2021 and 2024 from diverse clinical specimens, including urine (n = 74), blood (n = 11), pus (n = 21), exudates (n = 11), and other sample types (n = 28) at a diagnostic center in Chennai, India (Figure 1). Preliminary identification was performed based on colony morphology on MacConkey agar (HiMedia, India), where isolates typically appeared as large, mucoid, pink to red colonies. Species-level confirmation was achieved using the VITEK 2 automated system (bioMérieux). All isolates were preserved in cryoprotectant vials before subsequent phenotypic and genotypic analyses.”

A correction has been made to the section **Discussion**, *Therapeutic implications*. Remove- The removed phrase “nearly all β-lactams, including” overstated the resistance pattern; the corrected sentence provides a more accurate description.

“The coexistence of *bla*_NDM_ and *bla*_OXA-48, like_ in *K. pneumoniae*, represents a major therapeutic challenge, as these enzymes confer resistance to carbapenems. Current treatment options are largely confined to colistin, tigecycline, and ceftazidime–avibactam, although emerging resistance to the former two agents further limits efficacy. Combination regimens such as ceftazidime–avibactam plus aztreonam may offer improved activity against metallo-β-lactamase producers (Isler et al., 2022; Słabisz et al., 2024), while meropenem–vaborbactam and imipenem–relebactam demonstrate potential against OXA-48-like enzymes (Bonnin et al., 2022; Tiseo et al., 2024). Given these constraints, optimizing antibiotic stewardship, infection control, and rapid molecular diagnostics remains essential to guide targeted therapy and contain dissemination of these high-risk clones.”

The original version of this article has been updated.

