# Supplementary material for: Carbapenem-resistant Klebsiella pneumoniae from clinical infections: a multifactorial analysis of resistance, virulence, and biofilm potential
Source: Front Cell Infect Microbiol. 2025 Nov 24;15:1712034. doi: 10.3389/fcimb.2025.1712034 (PMC12682828; doi:10.3389/fcimb.2025.1712034)
Supplement: Supplementary file 1 [file Table1.docx]

Supplementary Material

**Carbapenem-resistant *Klebsiella pneumoniae* from clinical infections: A multifactorial analysis of resistance, virulence, and biofilm potential**

*Ramya Juliet^1^, Ramesh Nachimuthu^1^**

*^1^Antibiotic Resistance and Phage Therapy Laboratory, Centre for Advanced Research in Bacteriophage and Infectious Diseases, School of Bio Sciences and Technology, Vellore Institute of Technology (VIT), Vellore, 632014, Tamil Nadu, India*

**Correspondence:
Dr. Ramesh Nachimuthu: ramesh.n@vit.ac.in*

**Figure S1**

**MIC distribution of *K. pneumoniae* isolates to last-line antibiotics. Distribution of minimum inhibitory concentrations (MICs) for meropenem, colistin, and tigecycline among the 145 clinical *K. pneumoniae* isolates. Bars indicate the number of isolates at each MIC value.**


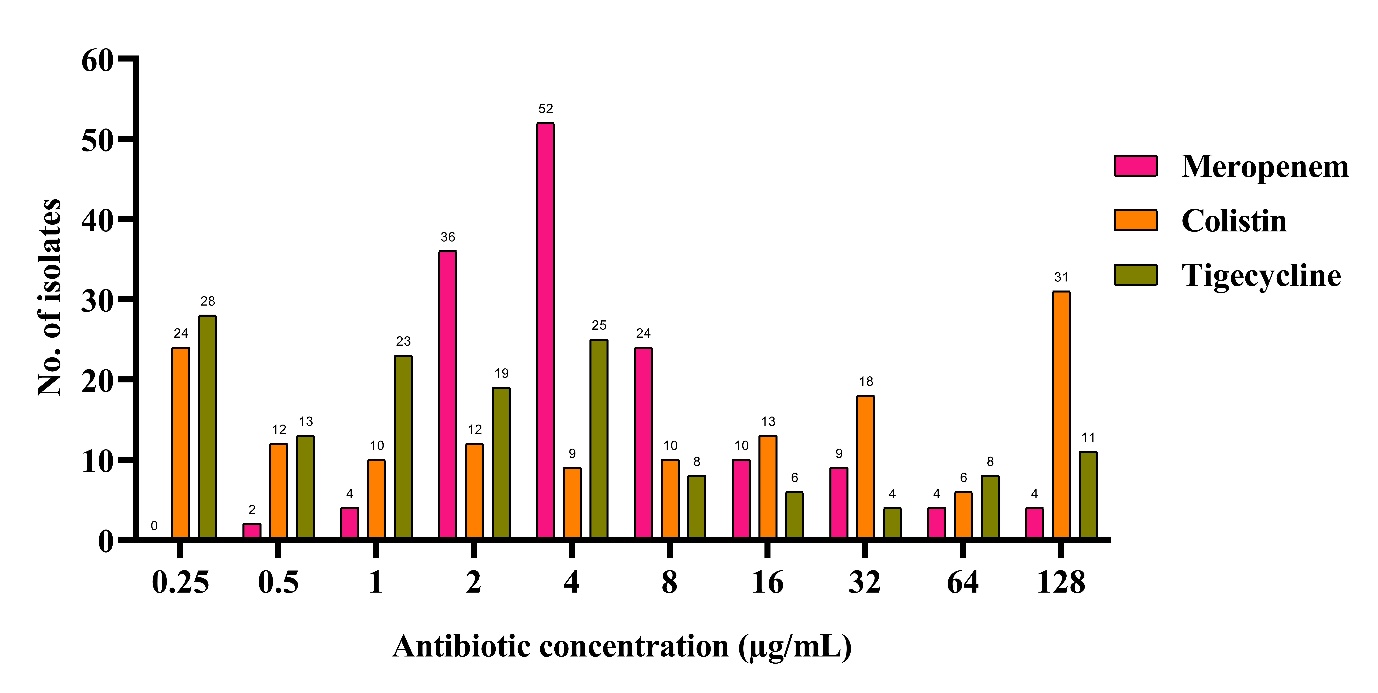


**Table S1.** The primers used for screening resistance and virulence genes are listed.

| **Primer** | **Primer sequence** | **Product size** | **Reference** |
| --- | --- | --- | --- |
| *bla*_KPC_ - F  *bla*_KPC_ - R | TGTCACTGTATCGCCGTC  CTCAGTGCTCTACAGAAAACC | 900 bp | (Manohar et al., 2021) |
| *bla*_IMP_ - F  *bla*_IMP_ - R | GAAGGCGTTTATGTTCATAC  GTACGTTTCAAGAGTGATGC | 587 bp |  |
| *bla*_VIM_ - F  *bla*_VIM_ - R | GTTTGGTCGCATATCGCAAC  AATGCGCAGCACCAGGATAG | 389 bp |  |
| *bla*_NDM_ - F  *bla*_NDM_ -R | GCAGCTTGTCGGCCATGCGGGC  GGTCGCGAAGCTGAGCACCGCAT | 782 bp |  |
| *bla*_OXA-48-like_ - F  *bla*_OXA-48-like_ - R | GCGTGGTTAAGGATGAACAC  CATCAAGTTCAACCCAACCG | 438 bp |  |
| Aerobactin-F  Aerobactin-R | GCATAGGCGGATACGAACAT  CACAGGGCAATTGCTTACCT | 556 bp | (Lin et al., 2014) |
| *rmpA-*F  *rmpA-*R | ACT GGG CTA CCT CTG CTT CA  CTT GCA TGA GCC ATC TTT CA | 535 bp |  |

**Table S2. Accession numbers of virulence genes aerobactin and *rmpA* submitted to NCBI**

| **Isolate ID** | **Gene** | **Accession no.** | **Gene** | **Accession no.** |
| --- | --- | --- | --- | --- |
| *K. pneumoniae* KP4 | Aerobactin | PP994840 | *rmpA* | PP994850 |
| *K. pneumoniae* KP12 | Aerobactin | PP994841 | *rmpA* | PP994851 |
| *K. pneumoniae* KP29 | Aerobactin | PP994842 | *rmpA* | PP994852 |
| *K. pneumoniae* KP30 | Aerobactin | PP994843 | *rmpA* | PP994853 |
| *K. pneumoniae* KP45 | Aerobactin | PP994844 | *rmpA* | PP994854 |
| *K. pneumoniae* KP64 | Aerobactin | PP994845 | *rmpA* | PQ066729 |
| *K. pneumoniae* KP66 | Aerobactin | PP994846 | *rmpA* | PP994855 |
| *K. pneumoniae* KP113 | Aerobactin | PP994847 | *rmpA* | PP994856 |
| *K. pneumoniae* KP115 | Aerobactin | PP994848 | *rmpA* | PP994857 |
| *K. pneumoniae* KP117 | Aerobactin | PP994849 | *rmpA* | PP994858 |

**Table S3. Accession numbers of resistance gene *bla*_OXA-48 like_ submitted to NCBI**

| Isolate ID | Gene | Accession no. |
| --- | --- | --- |
| *K. pneumoniae* KP13 | *bla*_OXA-48 like_ | PP746577 |
| *K. pneumoniae* KP26 | *bla*_OXA-48 like_ | PP746578 |
| *K. pneumoniae* KP35 | *bla*_OXA-48 like_ | PP770611 |
| *K. pneumoniae* KP36 | *bla*_OXA-48 like_ | PP746579 |
| *K. pneumoniae* KP37 | *bla*_OXA-48 like_ | PP746580 |
| *K. pneumoniae* KP39 | *bla*_OXA-48 like_ | PQ066732 |
| *K. pneumoniae* KP41 | *bla*_OXA-48 like_ | PP770609 |
| *K. pneumoniae* KP43 | *bla*_OXA-48 like_ | PP770612 |
| *K. pneumoniae* KP49 | *bla*_OXA-48 like_ | PP770610 |
| *K. pneumoniae* KP90 | *bla*_OXA-48 like_ | PP770607 |
| *K. pneumoniae* KP106 | *bla*_OXA-48 like_ | PP770608 |
| *K. pneumoniae* KP110 | *bla*_OXA-48 like_ | PQ066730 |
| *K. pneumoniae* KP111 | *bla*_OXA-48 like_ | PQ066731 |
| *K. pneumoniae* KP127 | *bla*_OXA-48 like_ | PP770606 |

**Table S4. Accession numbers of resistance gene *bla*_NDM_ submitted to NCBI**

| Isolate ID | Gene | Accession no. |
| --- | --- | --- |
| *K. pneumoniae* KP35 | *bla*_NDM_ | PP746576 |
| *K. pneumoniae* KP38 | *bla*_NDM_ | PP746575 |
| *K. pneumoniae* KP42 | *bla*_NDM_ | PP746574 |
| *K. pneumoniae* KP43 | *bla*_NDM_ | PP746573 |
| *K. pneumoniae* KP53 | *bla*_NDM_ | PP746572 |
| *K. pneumoniae* KP54 | *bla*_NDM_ | PP737686 |
| *K. pneumoniae* KP56 | *bla*_NDM_ | PP737687 |
| *K. pneumoniae* KP72 | *bla*_NDM_ | PP737688 |
| *K. pneumoniae* KP74 | *bla*_NDM_ | PP737689 |

**Table S5. Year-wise distribution of carbapenem-resistant and multidrug-resistant *K. pneumoniae* clinical isolates collected between 2021 and 2024.**

| **Year** | **No of isolates** | **CR-Kp** | **MDR-Kp** |
| --- | --- | --- | --- |
| 2021 | 25 | 21 (84%) | 18 (72%) |
| 2022 | 30 | 18 (60%) | 25 (83%) |
| 2023 | 45 | 37 (82%) | 35 (77%) |
| 2024 | 45 | 27 (60%) | 28 (62%) |
| **Total** | **145** | **103** | **106** |
